# Metabolites of the Nitric Oxide (NO) Pathway Are Altered and Indicative of Reduced NO and Arginine Bioavailability in Patients with Cardiometabolic Diseases Complicated with Chronic Wounds of Lower Extremities: Targeted Metabolomics Approach (LC-MS/MS)

**DOI:** 10.1155/2019/5965721

**Published:** 2019-07-14

**Authors:** Małgorzata Krzystek-Korpacka, Jerzy Wiśniewski, Mariusz G. Fleszar, Iwona Bednarz-Misa, Agnieszka Bronowicka-Szydełko, Małgorzata Gacka, Leszek Masłowski, Krzysztof Kędzior, Wojciech Witkiewicz, Andrzej Gamian

**Affiliations:** ^1^Department of Medical Biochemistry, Wroclaw Medical University, Wroclaw 50-368, Poland; ^2^PORT Polski Ośrodek Rozwoju Technologii sp, ZOO, Wroclaw 54-066, Poland; ^3^Department of Angiology, Hypertension and Diabetes, Wroclaw Medical University, Wroclaw 50-556, Poland; ^4^Department of Angiology, Regional Specialist Hospital, Wroclaw 51-124, Poland; ^5^Department of Vascular Surgery, Regional Specialist Hospital, Wroclaw 51-124, Poland; ^6^Research and Development Centre, Regional Specialist Hospital, Wroclaw 51-124, Poland

## Abstract

**Objective:**

The status of metabolites of the nitric oxide (NO) pathway in patients with chronic wounds in the course of cardiometabolic diseases is largely unknown. Yet arginine supplementation and citrulline supplementation as novel therapeutic modalities aimed at increasing NO are tested.

**Material and Methods:**

Targeted metabolomics approach (LC-MS/MS) was applied to determine the concentrations of L-arginine, L-citrulline, asymmetric and symmetric dimethylarginines (ADMA and SDMA), and arginine/ADMA and arginine/SDMA ratios as surrogate markers of NO and arginine availability in ulnar and femoral veins, representing systemic and local levels of metabolites, in patients with chronic wounds in the course of cardiometabolic diseases (*n* = 59) as compared to patients without chronic wounds but with similar cardiometabolic burden (*n* = 55) and healthy individuals (*n* = 88).

**Results:**

Patients with chronic wounds had significantly lower systemic L-citrulline and higher ADMA and SDMA concentrations and lower L-arginine/ADMA and L-arginine/SDMA as compared to healthy controls. The presence of chronic wounds in patients with cardiometabolic diseases was associated with decreased L-arginine but with increased L-citrulline, ADMA, and SDMA concentrations and decreased L-arginine/ADMA and L-arginine/SDMA. Serum obtained from the ulnar and femoral veins of patients with chronic wounds differed by L-arginine concentrations and L-arginine/SDMA ratio, both lower in the femoral vein. Wound etiology affected L-citrulline and SDMA concentrations, lower and higher, respectively, in patients with venous stasis, and the L-arginine/SDMA ratio—lower in venous stasis. The wound type affected L-arginine/ADMA and citrulline—lower in patients with ulcerations or gangrene. IL-6 was an independent predictor of L-arginine/ADMA, VEGF-A of ADMA, G-CSF of L-arginine/SDMA, and GM-CSF of L-citrulline and SDMA.

**Conclusion:**

Chronic wounds in the course of cardiometabolic diseases are associated with reduced NO and arginine availability due to ADMA and SDMA accumulation rather than arginine deficiency, not supporting its supplementation. Wound character seems to affect NO bioavailability and wound etiology—arginine bioavailability. Arginine concentration and its availability are more markedly reduced at the local level than the systemic level.

## 1. Introduction

Sufficient synthesis and bioavailability of nitric oxide (NO)—a free radical and a key vasodilator—are crucial for proper functioning of the vascular endothelium. Consequently, NO deficiency is a prerequisite for and a hallmark of endothelial dysfunction, a pathology preceding the development of cardiovascular diseases (CVD) [1]. CVD and their main risk factors, such as obesity, hypertension, and type 2 diabetes mellitus (T2DM), are, in turn, among key factors negatively affecting proper wound healing [2]. Nonhealing wounds constitute a serious problem for affected people and a growing burden for public health care [3]. Currently, they affect 4.5 million people in the United States alone [4] but the incidence of chronic wounds is likely to increase along with estimated rise in the incidence of CVD, obesity, and T2DM [5–8]. The overall prevalence of peripheral artery disease (PAD) in Europe is estimated to be 5.3% but differs by country [9]. The disturbed blood flow and blood vessel damage accompanying CVD and specifically PAD may result in ulcerations or gangrene located in the lower extremities [9–11]. NO-releasing wound dressings as well as diet supplementation with L-arginine, a NO substrate, are currently being evaluated as novel modalities in the treatment of chronic wounds [12, 13].

Successful healing requires spacial and temporal cooperation of a myriad of players, mediating three key phases of the process: inflammatory, proliferative, and remodelling [5]. Nonhealing wounds are believed to be locked in the inflammatory phase [14]. While most mediators are proteins, other molecules, such as NO, are recently gaining attention as potentially relevant for all phases of the healing [15–19]. The importance of NO has been demonstrated by the delayed healing of animals with genetically impaired NO synthesis [20, 21]. Moreover, it has been shown that NO therapy is effective in healing ischemic [22] and diabetic ulcers [23] in experimental animals by inducing reepithelization, angiogenesis, and collagen synthesis.

NO is synthesized by nitric oxide synthases (NOS) from L-arginine, and L-citrulline is the other reaction product. There are three isoforms of the enzyme: constitutively expressed endothelial (eNOS; NOS1) and neuronal (nNOS; NOS3) isoforms and the inducible isoform (iNOS; NOS2) [24]. The activity of NOS enzymes is regulated by methylated derivatives of arginine, of which asymmetric dimethylarginine (ADMA) is believed to be a strong and symmetric dimethylarginine (SDMA) a weak enzyme inhibitor. Both ADMA and SDMA compete with L-arginine for its transporters, and therefore, their accumulation decreases NO production by diminishing L-arginine availability for the NOS enzymes. The ADMA and SDMA pool is regulated at the level of their synthesis, conducted by the protein arginine methyltransferases (PRMTs), and degradation. While ADMA is mostly catabolized to L-citrulline and dimethylamine (DMA), by dimethylarginine dimethylaminohydrolases (DDAHs), SDMA is preferentially excreted with urine. L-Citrulline may be used in the arginine-citrulline cycle to satisfy the body demand for L-arginine [24].

The gaseous nature, high diffusion capacity, and short half-life make NO the ideal signalling molecule but cause its quantification to be a challenge. Therefore, relatively more stable products of NO oxidation, nitrites and nitrates, are measured instead of NO. Nitrate has even been proposed as a “wound healing biomarker and surrogate end point” for treatment of diabetic foot ulcers [18]. Another approach for the assessment of NO and its bioavailability is the evaluation of intermediates in the NO synthesis pathway and inhibitors of NOS enzymes. Of those, the measurement of L-arginine and/or ADMA is the most popular.

Despite the relevance of NO for wound healing, the status of its pathway metabolites and surrogate markers is largely unknown. Recently, an elevation in serum ADMA [25] has been reported in patients with chronic wounds while our own preliminary research showed a decrease in serum L-arginine [26]. However, there seem to be a paucity of data on SDMA and citrulline or regarding the possible association between NO metabolites and wound etiology and the type or their interplay with inflammatory and immune mediators. Therefore, this study was designed to simultaneously evaluate a wider panel of L-arginine/NO pathway metabolites using targeted metabolomics approach and a novel assay recently developed by our group [27]. We measured L-arginine, L-citrulline (referred to hereafter simply as arginine and citrulline), ADMA, and SDMA to determine their status and clinical relevance in patients with chronic wounds of various etiologies and types, at systemic and local levels.

## 2. Materials and Methods

### 2.1. Ethical Approval

The study protocol was approved by the Medical Ethics Committees of Wroclaw Medical University (#KB-384/2012) from April 12, 2012, and the study was conducted in accordance with the Helsinki Declaration of 1975, as revised in 1983, and an informed consent has been obtained from all patients.

### 2.2. Patients

The study population consisted of 202 individuals: 114 patients with cardiovascular diseases and/or diabetes, of whom 59 had chronic wounds of lower extremities, and 88 healthy individuals. Patients were recruited from among patients of the Dept. of Angiology of the Regional Specialist Hospital and the Dept. of Angiology, Hypertension and Diabetes of the Wroclaw Medical University. Concerning patients with nonhealing wounds, only those with wounds in the course of vascular disease and diabetes were included while others, with wounds due to autoimmune diseases, malignancy, infections, or drugs, were not enrolled. Wound etiology was determined by the evaluation of wound characteristics (location and an appearance of the wound, its borders and those of the surrounding skin, pain, and the presence of bleeding on manipulation) in addition to the patient's history and clinical assessment, which was based on the ankle-brachial pressure index, ultrasound, angiography, and computer tomography. The wound etiology was as follows: venous stasis (*n* = 25), ischemic (arterial) (*n* = 17), and neurotrophic (*n* = 17). Patients' wounds were mechanically cleaned of necrotic tissue and excess wound exudate, photographs were taken, and wound material was collected for bacteriological examination. Subsequently, wounds were washed with antiseptic octenidine dihydrochloride and dressed using a sterile gauze. None of the patients used arginine supplements.

**Figure 1:**
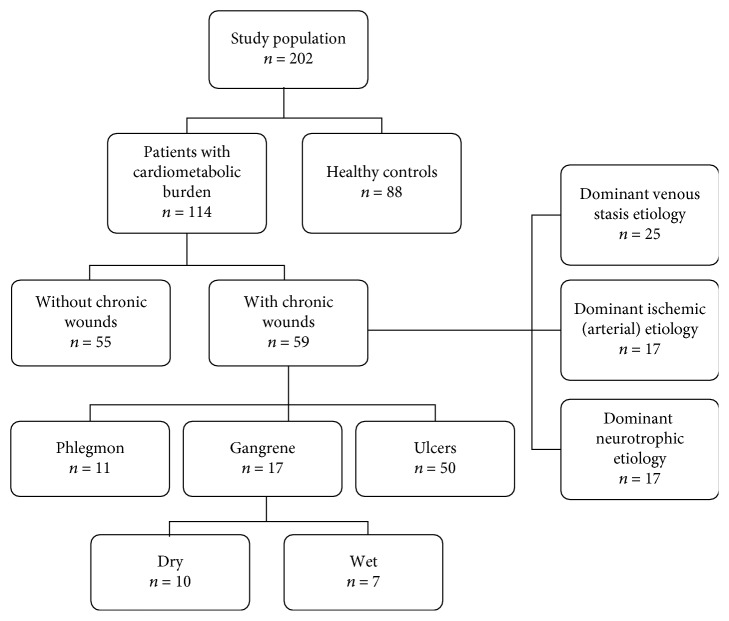
Diagram showing the categorization of study population into groups.

The control group consisted of healthy volunteers recruited from hospital staff and blood donors from Lower Silesian Center of Blood Donation and Therapy, Wroclaw, Poland (recruited on the basis of standard eligibility criteria for blood donation with an age > 50 yrs. and an insignificant medical history as additional inclusion criteria for the current study).  Table 1 presents detailed characteristics of the study population, and the categorization into examined groups is depicted in Figure 1.

In a subgroup of patients (*n* = 7), blood was sampled from the ulnar and femoral veins in order to compare systemic metabolite concentrations with those more local and closer to the wound.

### 2.3. Analytical Methods

Blood (7.5 mL), after an overnight fasting, was drawn into serum separator tubes from ulnar veins and, additionally, from femoral veins. Blood was clotted for 30 min and subsequently centrifuged (15 min, 10°C, 720×*g*). Collected serum was aliquoted and kept frozen at -80° until examination.

#### 2.3.1. Materials

LC-MS-grade acetonitrile, water, and methanol were purchased from Merck Millipore (Warsaw, Poland). L-Arginine, SDMA, ADMA, L-citrulline, sodium tetraborate, benzoyl chloride (BCl), and HPLC-grade formic acid (FA) were obtained from Sigma-Aldrich (Poznan, Poland). Isotope-labeled asymmetric dimethylarginine (2,3,3,4,4,5,5-D7-ADMA, 98%) and L-arginine:HCl (D7-arginine, 98%) were acquired from Cambridge Isotope Laboratories (MA, USA). Leucine-enkephalin was purchased from Waters (Warsaw, Poland).

#### 2.3.2. Quantitative Analysis of Metabolites Involved in NO Synthesis

Serum concentrations of metabolites involved in NO synthesis were measured by stable isotope dilution liquid chromatography tandem mass spectrometry using a Xevo G2 quadrupole-TOF instrument (Waters, Milford, MA, USA) as described in detail by Fleszar et al. [27]. Briefly, aliquots of 100 *μ*L of sera or the calibration sample, 10 *μ*L of internal standard solution in water (100 *μ*M D7-arginine and 20 *μ*M D7-ADMA), and 50 *μ*L of borate buffer (0.025 M Na_2_B_4_O_7_·10H_2_O, 1.77 mM NaOH, pH = 9.2) were transferred into polypropylene microtubes and vortexed (1 min, 1100 rpm, 25°C). Then, 400 *μ*L of acetonitrile and 10 *μ*L of 10% BCl in acetonitrile were added and vortexed (5 min, 1100 rpm, 25°C). After derivatization, samples were centrifuged (7 min, 4°C, 15000 × *g*) and 100 *μ*L of supernatants was transferred into a chromatographic glass vial with 300 *μ*L of water for LC-MS analysis.

#### 2.3.3. LC-ESI-MS Analysis

The LC analysis was carried out on a nanoACQUITY UPLC System equipped with an ACQUITY HSS T3 column (50 × 1.0 mm, 1.75 *μ*m) with a 0.22 *μ*m membrane inline filter (Waters). The total run time of the method was 10 min with a flow rate of 80 *μ*L/min. Mobile phase A consisted of 0.1% FA in water, while mobile phase B consisted of 0.1% FA in methanol. For ADMA and SDMA isomer separation, the following gradient was applied: 11% B for 0–1 min, 11%–13% B for 1–2 min, 13%–60% B for 2–5 min, 60%–90% B for 5–5.5 min, 90% B for 5.5–6 min, and 90%–11% B for 6–6.05 min. The sample injection volume was 2 *μ*L.

Mass spectra for the compounds were acquired in a Xevo G2 Q-TOF mass spectrometer (Waters) in positive ion mode electrospray ionization (ESI). The MS operating conditions were as follows: capillary voltage, 3000 V; cone voltage, 40 V; source temperature, 120°C; cone gas flow, 85 L/hour; desolvation temperature, 350°C; and desolvation gas flow, 800 L/hour. Data acquisition was carried on MassLynx Software (Waters) using the following ions: 279.1457 *m*/*z*, 286.1897 *m*/*z*, 307.1770 *m*/*z*, 314.2076 *m*/*z*, and 280.1136 *m*/*z* for L-arginine, D7-arginine, ADMA and SDMA, D7-ADMA, and L-citrulline, respectively.

As previously described [27], the method is characterized by intra- and interassay coefficients of variation of 1.6% and 3.3% for arginine, 3.2% and 3.1% for citrulline, 7.5% and 9.4% for ADMA, and 6.4% and 7.1% for SDMA determination.

### 2.4. Statistical Analysis

Data were tested for normality of distribution using the Kolmogorov-Smirnoff test and for homogeneity of variances using Levene's test and presented as means or medians with 95% confidence interval (CI) around them. Between-group differences in means or medians were tested using a *t*-test for independent samples with Welch correction if appropriate or with the Mann-Whitney *U* test (two-group comparisons) and with a one-way ANOVA with the Tukey-Kramer post hoc test or with the Kruskal-Wallis *H* test with the Conover post hoc test (multigroup comparisons). Log-transformation was used if necessary to obtain normality of distribution and/or homogeneity of variances. Additionally, a *t*-test for paired samples was used to analyze differences in metabolite concentrations between femoral and ulnar veins. Frequency analysis was conducted using the chi-squared test. Univariate correlations were examined using Pearson tests. Multivariate linear regression was conducted to discern the independent predictor of NO-associated metabolites. Regression models were built with a stepwise method using the following criteria: enter variable if *p* < 0.05 and remove variable if *p* > 0.1. All calculated probabilities were two-tailed, and *p* values ≤ 0.05 were considered statistically significant. The analyses were performed using MedCalc Statistical Software version 18.11.6 (MedCalc Software bvba, Ostend, Belgium; https://www.medcalc.org; 2019).

## 3. Results

Both patients' cohorts were well matched with respect to sex distribution, age, and concentrations of biochemical indices, indicative of similar disease burden. There were, however, significant differences in HDL cholesterol and CRP lower and higher, respectively, in patients with chronic wounds (Table 1). There were no sex-related differences in any intermediate in the arginine/NO pathway among patients with chronic wounds or healthy controls. However, in a group of patients with cardiometabolic diseases without wounds, males had higher citrulline, ADMA, and SDMA concentrations and lower Arg/ADMA and Arg/SDMA ratios (for details, see Suppl. Tab.  1).

### 3.1. Intermediates in the Arginine/NO Pathway in Patients with Cardiometabolic Diseases with and without Chronic Wounds

**Figure 2:**
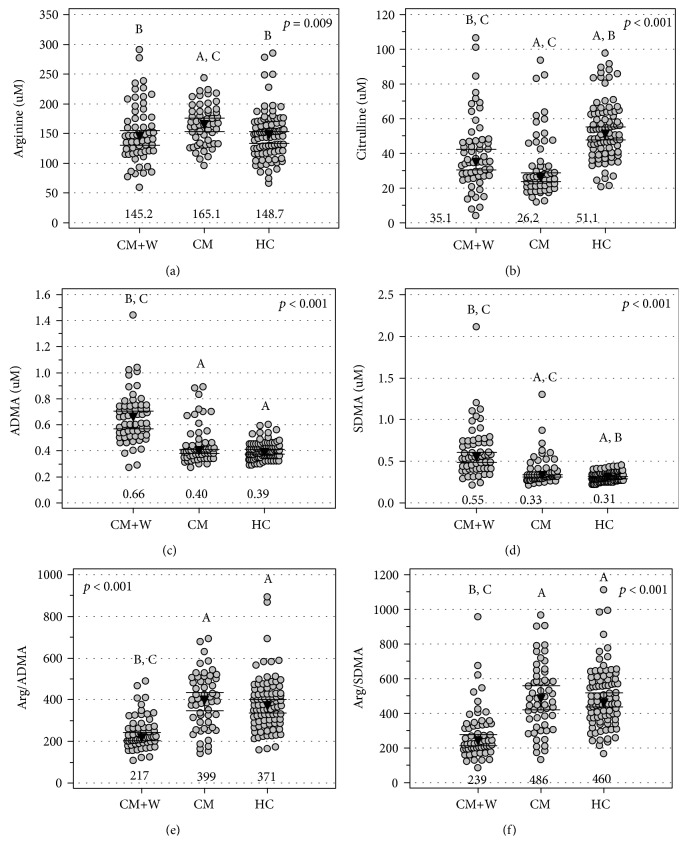
Intermediates in the arginine/NO pathway in chronic wounds: (a) arginine, (b) citrulline, (c) ADMA, (d) SDMA, (e) arginine-to-ADMA ratio (Arg/ADMA), and (f) arginine-to-SDMA ratio (Arg/SDMA). Data are presented as medians with 95% confidence intervals and analyzed using the Kruskal-Wallis *H* test. CM+W: patients with cardiometabolic diseases and chronic wounds; CM: patients with cardiometabolic diseases without chronic wounds; HC: healthy controls. Numbers below the dot plots represent the mean value within a group. Letters above the dot plots indicate groups from which a given group mean differs significantly: A: significantly different from CM+V; B: significantly different from CM; C: significantly different from HC.

As compared to healthy individuals, patients with cardiometabolic diseases without chronic wounds had significantly higher arginine (Figure 2(a)) but lower citrulline (Figure 2(b)) concentrations and comparable ADMA and SDMA concentrations (Figures 2(c) and 2(d)) and Arg/ADMA and Arg/SDMA ratios (Figures 2(e) and 2(f)). As compared to healthy individuals, patients with cardiometabolic diseases with chronic wounds had significantly lower citrulline (Figure 2(b)) and higher ADMA and SDMA concentrations (Figures 2(c) and 2(d)) and lower Arg/ADMA and Arg/SDMA ratios (Figures 2(e) and 2(f)). The presence of chronic wounds in patients with cardiometabolic diseases was associated with decreased arginine (Figure 2(a)) but with increased citrulline (Figure 2(b)), ADMA, and SDMA concentrations (Figures 2(c) and 2(d)) and decreased Arg/ADMA and Arg/SDMA ratios (Figures 2(e) and 2(f)).

### 3.2. Impact of Wound Etiology on Intermediates in the Arginine/NO Pathway

**Figure 3:**
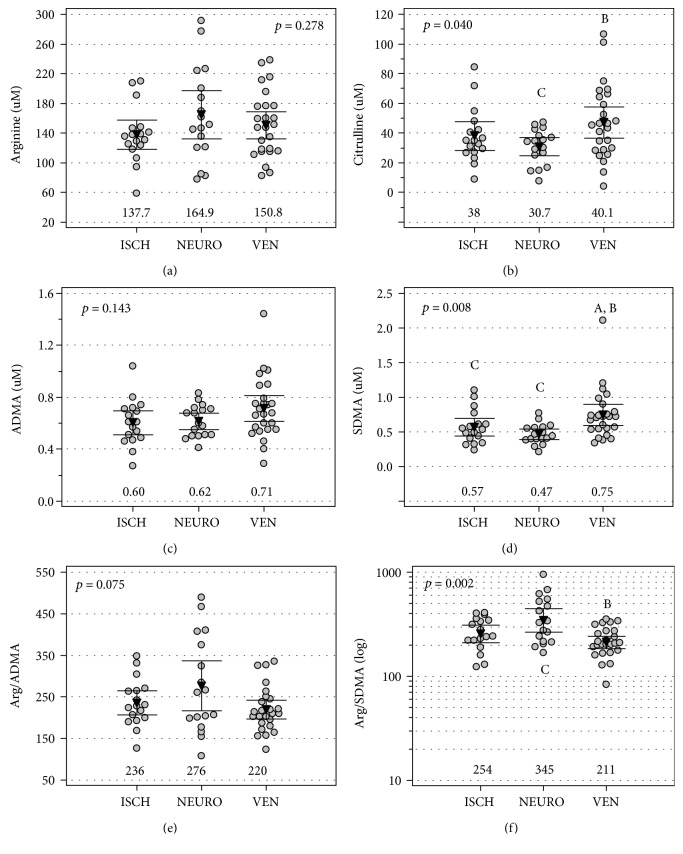
Wound etiology and intermediates in the arginine/NO pathway: (a) arginine, (b) citrulline, (c) ADMA, (d) SDMA, (e) arginine-to-ADMA ratio (Arg/ADMA), and (f) arginine-to-SDMA ratio (Arg/SDMA). Data are presented as means with 95% confidence intervals and analyzed using one-way ANOVA. ISCH: ischemic etiology; NEURO: neurotrophic etiology; VEN: venous stasis etiology. Numbers below the dot plots represent the mean value within a group. Letters above/below the dot plots indicate groups from which a given group mean differs significantly: A: significantly different from ISCH; B: significantly different from NEURO; C: significantly different from VEN.

Arginine (Figure 3(a)) and ADMA (Figure 3(c)) did not differ with respect to wound etiology, but citrulline concentrations (Figure 2(b)) were significantly higher in patients with venous stasis wounds than in those with neurotrophic wounds. SDMA concentrations (Figure 3(d)) were significantly higher in patients with venous stasis wounds as compared to both neurotrophic and ischemic wounds. The Arg/SDMA ratio (Figure 2(e)) was significantly lower in patients with venous stasis wounds as compared to neurotrophic wounds, and the Arg/ADMA ratio displayed a similar tendency (Figure 3(f)).

### 3.3. Intermediates in the Arginine/NO Pathway and Wound Characteristics

The concentrations of intermediates of the arginine/NO pathway were compared between patients with and without gangrene and between two of its types (dry and wet) as well as between patients with and without phlegmon or with and without ulceration.

**Figure 4:**
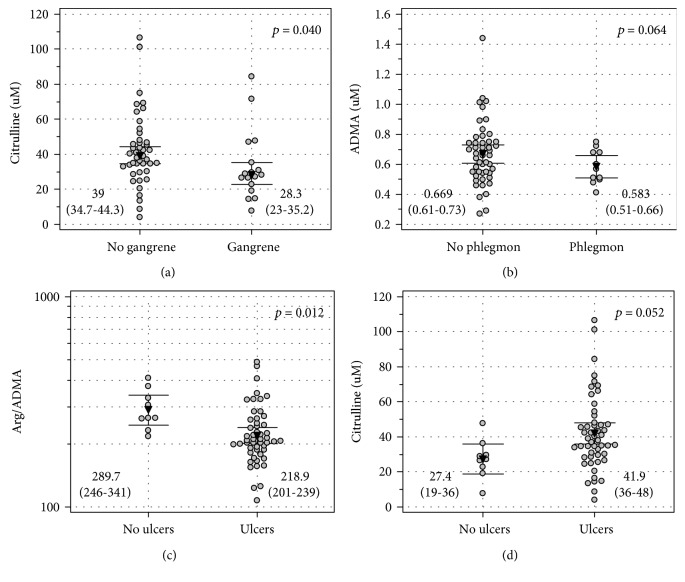
Wound type and intermediates in the arginine/NO pathway: (a) citrulline and gangrene, (b) ADMA and phlegmon, (c) Arg/ADMA and ulceration, and (d) citrulline and ulceration. Data are presented as (a) medians or means with 95% confidence intervals and analyzed using (a) the Mann-Whitney *U* test or *t*-test for independent samples.

Concerning gangrene, only citrulline concentrations differed significantly and were lower in patients with gangrene (Figure 4(a)). There were no differences in other intermediates in the arginine/NO pathway with respect to the presence or absence of gangrene (*p* = 0.443 for arginine, *p* = 0.327 for ADMA, *p* = 0.775 for SDMA, *p* = 0.159 for Arg/ADMA, and *p* = 0.547 for Arg/SDMA) (for details, see Suppl. Fig.  1). Also, the intermediates did not differ with respect to the gangrene type (*p* = 0.905 for arginine, *p* = 0.508 for citrulline, *p* = 0.990 for ADMA, *p* = 0.810 for SDMA, *p* = 0.859 for Arg/ADMA, and *p* = 0.940 for Arg/SDMA) (for details, see Suppl. Fig.  2).

Concerning phlegmon, there were no differences as well (*p* = 0.599 for arginine, *p* = 0.183 for citrulline, *p* = 0.134 for SDMA, *p* = 0.208 for Arg/ADMA, and *p* = 0.231 for Arg/SDMA) (for details, see Suppl. Fig.  3), although ADMA tended to be lower in patients with phlegmon (Figure 4(b)).

Concerning ulceration, ADMA (*p* = 0.118) only tended to be higher in patients with ulcers and arginine (*p* = 0.291) tended to be lower (for details, see Suppl. Fig.  4) but their ratio (Arg/ADMA) was significantly lower in patients with ulceration as compared to those without (Figure 4(c)). Citrulline tended to be elevated in patients with ulceration (Figure 4(d); 41.9 *μ*M (35.9-47.9) vs. 27.4 *μ*M (18.8-35.9), *p* = 0.052), and SDMA (*p* = 0.351) and Arg/SDMA (*p* = 0.101) did not show significant differences (for details, see Suppl. Fig.  4).

### 3.4. Intermediates in the Arginine/NO Pathway and Blood Source

**Figure 5:**
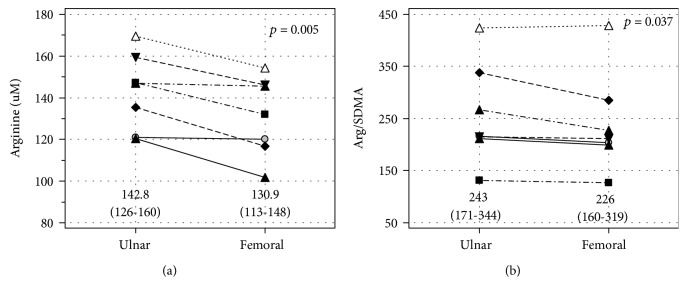
Difference in (a) arginine concentrations and (b) arginine-to-SDMA ratio between the ulnar and femoral veins. Data are presented as means with 95% confidence intervals and analyzed using a *t*-test for paired samples.

For a subgroup of seven patients, blood was sampled from both the ulnar and femoral veins. The comparison of metabolite concentrations with respect to blood source did not show statistically significant differences for ADMA (*p* = 0.852), SDMA (*p* = 0.554), Arg/ADMA (*p* = 0.198), and citrulline (*p* = 0.954) (for details, see Suppl. Fig.  5). However, concentrations of arginine (Figure 5(a)) as well as Arg/SDMA (Figure 5(b)) were significantly lower in blood sampled from the femoral vein than the ulnar vein.

### 3.5. Correlation Pattern between Intermediates in the Arginine/NO Pathway and Cytokines, Chemokines, and Growth Factors

The correlation patterns between intermediates in the arginine/NO pathway and key cytokines, chemokines, and growth factors implicated in the pathogenesis of wound healing have been examined (Table 2). There was no significant correlation between arginine and any of evaluated mediators. Citrulline correlated negatively with G-CSF, GM-CSF, IL-4, and IL-8. Of these, GM-CSF was an independent predictor of citrulline concentrations, explaining 15% in its variability (*r*_partial_ = −0.39, *p* = 0.013; *R* ^2^ = 0.155). ADMA correlated negatively with GM-CSF, TNF*α*, and VEGF-A. Of these, VEGF-A was an independent predictor of ADMA concentrations, explaining 13% in its variability (*r*_partial_ = −0.37, *p* = 0.022; *R* ^2^ = 0.134). SDMA correlated negatively with G-CSF, GM-CSF, IL-1*β*, IL-4, IL-8, and TNF*α*. Of these, GM-CSF was an independent predictor of SDMA concentrations, explaining 17% in its variability (*r*_partial_ = −0.42, *p* = 0.008; *R* ^2^ = 0.174). Arg/ADMA correlated with G-CSF, GM-CSF, IL-1*β*, IL-4, IL-6, VEGF-A, and TNF*α*, of which IL-6 was an independent predictor, explaining 28% of variability in its value (*r*_partial_ = 0.52, *p* < 0.001; *R* ^2^ = 0.275). Arg/SDMA correlated with G-CSF, GM-CSF, IL-1*β*, IL-4, and TNF*α*, of which G-CSF was an independent predictor of the Arg/SDMA ratio, explaining 17% in its variability (*r*_partial_ = 0.41, *p* = 0.010; *R* ^2^ = 0.167).

### 3.6. Interplay between Intermediates in the Arginine/NO Pathway

The pattern of interrelationships of intermediates in the arginine/NO pathway in patients with cardiometabolic diseases with and without chronic wounds has been examined. Arginine did not correlate with any other metabolite in patients with cardiometabolic diseases without chronic wounds but became directly correlated with the ADMA and SDMA levels in patients with chronic wounds. Citrulline was positively correlated with ADMA and SDMA in both patient groups, but the associations were weaker in patients with chronic wounds. ADMA correlated positively with SDMA, and the association was weaker in patients with chronic wounds (Table 3).

## 4. Discussion

Detrimental effects of diminished NO bioavailability on cardiovascular health and wound healing are well documented and have led to an outburst of novel treatment strategies aiming at its increase. Intuitively, an elevation in arginine, a direct substrate for NOS enzymes, ought to increase NO availability. Indeed, in addition to wound dressings containing NO precursors [12], the effect of arginine supplementation on a diet has been found beneficial for facilitating the healing of pressure ulcers [13]. However, our results do not confirm that systemic arginine is diminished in patients with chronic wounds to warrant its supplementation. Rather unexpectedly, they were increased in patients with cardiometabolic burden but without chronic wounds. This observation, however, agrees well with previously reported increased risk for CVD in patients with elevated arginine, independent from traditional risk factors [28]. Still, we also demonstrated that arginine concentrations in the blood from the femoral vein, draining the wounded leg, are significantly lower implying that arginine might indeed be depleted locally. Moreover, also the Arg/SDMA ratio was markedly reduced in the femoral vein than in the ulnar vein, indicative of more severe local arginine depletion.

Nonetheless, it has been suggested that an elevation in systemic or local arginine may not directly translate into increased NO concentrations, a phenomenon explained by impaired arginine availability for NO synthesis by NOS enzymes. At least several mechanisms are in operation, that is, concomitant upregulation of NOS inhibitors, NOS uncoupling due to oxidative stress and tetrahydrobiopterin deficiency, and arginine utilization by an upregulated arginase [29]. Indeed, corroborating the first mechanism, we showed that patients with chronic wounds had increased both ADMA and SDMA concentrations as compared to healthy individuals and patients with cardiometabolic diseases without chronic wounds. Elevated ADMA increases cardiovascular risk [30] and is associated with every disease within the CVD spectrum as well as with CVD risk factors [31]. Mechanistically, ADMA interferes with NO synthesis by inhibiting NOS enzymes and by reducing arginine availability by competing for its membrane transporters. Additionally, it impairs NO signalling by inhibiting eNOS phosphorylation [32]. Also, El-Mesallamy et al. [25] demonstrated that ADMA was elevated in patients with leg ulcers significantly more so than in T2DM patients without neuropathy. SDMA negatively affects arginine availability by inhibiting its membrane transport as well but has not gained as much attention as ADMA as it is only a weak NOS inhibitor. There seems to be a paucity of information on SDMA in chronic wounds in the course of cardiometabolic diseases. In turn, data linking SDMA with a risk of CVD and CVD-caused mortality derived from meta-analyses are contradictory [30, 33]. However, individual studies indicate SDMA elevation to be an independent predictor of CVD-related mortality [34, 35] and to predict renal and cardiovascular outcomes in patients with chronic kidney disease [36]. Furthermore, functional studies have shown SDMA to abolish anti-inflammatory and antiatherogenic properties of HDL. Consequently, SDMA has been claimed to be a marker of HDL dysfunction [34].

We also observed that due to ADMA and SDMA accumulation, both Arg/ADMA and Arg/SDMA ratios were significantly decreased in patients with chronic wounds, indicating reduced availability of NO and arginine, respectively. Wound character seemed to have an impact on NO bioavailability since the Arg/ADMA ratio was markedly reduced in patients with ulcerations. In turn, wound etiology affected arginine bioavailability as patients with venous stasis had markedly elevated SDMA and decreased Arg/SDMA. The ADMA and SDMA pool is regulated mainly by the rates of their synthesis by type I (ADMA) and type II (SDMA) PRMTs and the rate of their degradation by DDAH enzymes (ADMA) and renal excretion [24]. Impaired renal secretion does not seem to explain the phenomenon of their accumulation in full as cardiometabolic burden, including chronic kidney disease, was similar in patients with and without chronic wounds. There is a paucity of data regarding PRMT and DDAH activity and expression in metabolic disorders and in chronic wounds. However, limited animal studies have linked PRMT overexpression with obesity, nonalcoholic fatty liver disease, and diabetic retinopathy [37, 38] and DDAH downregulation with diabetic retinopathy [38] and impaired vascular homeostasis [39]. PRMT and DDAH might be altered more strongly among patients with chronic wounds than without as they are positively (PRMTs) and negatively (DDAHs) affected by inflammatory mediators [31, 40] and, as we have previously demonstrated, chronic wounds are accompanied by systemic elevation in proinflammatory cytokines [41].

An interesting observation is the difference between SDMA, which was elevated in patients with cardiometabolic burden as compared to controls, and ADMA, which was not. It might result from differences in sensitivity of distinct PRMT enzymes. It has been shown that ADMA-yielding PRMT2 expression is inhibited by high glucose concentration, a common occurrence among our patients, which, in turn, upregulates SDMA synthesis owing to substrate scavenging by type II PRMTs [37].

A direct link between arginine and NO concentrations is further disturbed by NOS uncoupling [29]. The lack of arginine [42], high ADMA concentrations [43], inflammation and oxidative stress [44], and tetrahydrobiopterin deficiency [45] are considered main culprits of switching enzyme activity from NO synthesis to the production of superoxide anion. As such, the uncoupling of NOS leads not only to the concomitant decrease in NO availability but also to the exacerbation of oxidative stress. Furthermore, NOS enzymes compete for arginine with arginases, the enzymes converting arginine to ornithine. Correspondingly, the upregulated *ARG1* expression has been reported in patients with diabetic foot and venous ulcers [46] as well as in chronic wounds in recessive dystrophic epidermolysis bullosa [47].

Data regarding citrulline association with chronic wounds in the course of cardiometabolic diseases are missing, and those on citrulline in underlying conditions seem to be contradictory. Elevated citrulline has been associated with an increased CVD risk [28] and has a negative impact on arginine bioavailability in obesity and T2DM [48]. However, it is also an effective antioxidant [49], argued to be more effective in restoring cardiometabolic health *via* increasing NO availability than arginine [49]. In addition to improving endothelial function *via* NO-associated mechanisms, citrulline supplementation in patients with vasospastic angina has been shown to reduce the concentrations of oxidized LDL thus alleviating oxidative stress [50]. Citrulline in our study was diminished in patients with cardiometabolic burden as compared to healthy individuals and strongly and positively correlated with NOS inhibitors—ADMA and SDMA. Considering that citrulline is a second reaction product and an effective arginine precursor, these findings seem to confirm reduced rates of NO synthesis among patients with cardiometabolic burden and especially among patients with gangrene. Still, citrulline was less diminished in patients with chronic wounds than those without, particularly in case of wounds of venous stasis etiology. Also, in line with its negative impact on arginine availability, it negatively and strongly correlated with the Arg/SDMA ratio. Debats et al. [51], in turn, found citrulline to be elevated exclusively in patients with infected chronic wounds but not with noninfected or acute wounds.

Inflammatory cytokines are among the initiators of endothelial dysfunction [44] and key players in sustaining inflammation in chronic wounds. Among others, they induce the expression of iNOS [16] but inhibit that of eNOS [52, 53] and contribute to ADMA accumulation [31, 40]. Recently, we have demonstrated that chronic wounds are accompanied by systemic elevation of IL-1*β*, IL-4, IL-6, IL-8, FGF-2, MIP-1*α*, PDGF-BB, and VEGF-A [41]. Also, markedly elevated CRP and reduced HDL in patients with chronic wounds compared to those with similar cardiometabolic burden are indicative of a higher grade of inflammation in the former. Among our patients, ADMA concentrations were independently and inversely associated with VEGF-A, indicative of the negative impact of ADMA accumulation and resulting diminished NO synthesis on angiogenesis in patients with chronic wounds. We also observed that the higher inflammatory response, indicated by IL-6 concentration, the lower the NO availability, indicated by the reduced Arg/ADMA ratio. We found that GM-CSF was an independent predictor for citrulline and SDMA and G-CSF for Arg/SDMA. G-CSF and GM-CSF are hematopoietic cytokines displaying immunomodulatory and antibiotic-enhancing activities with a proven beneficial effect on wound healing [54–56]. Their concentrations are reduced in patients with chronic wounds [41]. Park et al. [57] demonstrated that G-CSF exerts a protective effect on endothelial cells *via* stimulating eNOS expression and phosphorylation and thus enhancing NO synthesis and signalling. The close relation between G-CSF and Arg/SDMA observed here may indicate an additional mechanism, that is, increased arginine availability.

## 5. Conclusions

Taken together, our results demonstrate that patients with chronic wounds in the course of cardiometabolic diseases have reduced bioavailability of NO and its substrate, arginine, resulting from ADMA and SDMA accumulation rather than from arginine deficiency. Citrulline, in turn, is decreased in patients with cardiometabolic diseases in general, but the presence of chronic wounds is associated with its elevation, reflecting degree of ADMA, and SDMA accumulation and inversely related to NO and arginine bioavailability. As such, our findings do not support arginine or citrulline supplementation in patients with chronic wounds and rather suggest the need for treatment aiming at decreasing ADMA and SDMA concentrations.

## Figures and Tables

**Table 1:** Characteristics of the study population.

Parameter	Healthy controls	Patients with cardiometabolic diseases		*p* value
		Without chronic wounds	With chronic wounds	
Number of patients	88	55	59	—
Age (yrs.), median (range)	63 (50-73)	64 (49-81)	65 (40-87)	0.086^K^
Sex (F/M), *n*	33/55	29/26	22/37	0.145^*χ*2^
FG (mg/dL), mean (95% CI)	—	159.5 (149-179)	147.2 (105-189)	0.591^W^
HbA1C (%), mean (95% CI)	—	7.73 (7.2-8.2)	7.98 (7-8.9)	0.620^t^
CHOL (mg/dL), median (95% CI)	—	168 (163-177)	164 (147-175)	0.162^M^
HDL (mg/dL), median (95% CI)	—	44.5 (40-49)	35 (32-44)	0.008^M^
LDL (mg/dL), mean (95% CI)	—	101 (91-112)	89.8 (81-99)	0.104^W^
TG (mg/dL), mean (95% CI)	—	148.4 (129-171)	126.7 (108-148)	0.156^t^
Creatinine (mg/dL), mean (95% CI)	—	0.98 (0.92-1.04)	0.89 (0.78-1.02)	0.191^W^
HGB (g/dL), mean (95% CI)	—	13.5 (13-13.9)	12.9 (12.4-13.8)	0.084^t^
CRP (mg/L), mean (95% CI)	—	1.7 (1.3-2.3)	19.6 (13.5-28.5)	<0.0001^t^

yrs.: years; F/M: female-to-male ratio; SD: standard deviation; *n*: number of patients; FG: fasting glucose; HbA1C: glycated hemoglobin; CHOL: total cholesterol; HDL: high-density lipoprotein cholesterol; LDL: low-density lipoprotein cholesterol; TG: triglycerides; HGB: hemoglobin; CRP: C-reactive protein; ^K^Kruskal-Wallis *H* test; ^W^Welch test; ^*χ*2^chi-squared test; ^t^ *t*-test for independent samples; ^M^Mann-Whitney *U* test.

**Table 2:** Correlation pattern between intermediates in the arginine/NO pathway and cytokines, chemokines, and growth factors.

Cytokine	Arg	Cit	ADMA	SDMA (log)	Arg/ADMA	Arg/SDMA
FGF2	0.13	-0.29	-0.17	-0.28	0.21	0.27
G-CSF	0.12	-0.35^∗^	-0.27	-0.37^∗^	0.42^‡^	0.41^‡^
GM-CSF (log)	0.06	-0.39^∗^	-0.36^∗^	-0.42^‡^	0.39^∗^	0.36^∗^
IL-1*β*	0.10	-0.27	-0.27	-0.36^∗^	0.38^∗^	0.35^∗^
IL-4	0.17	-0.32^∗^	-0.21	-0.34^∗^	0.32^∗^	0.36^∗^
IL-6 (log)	0.08	-0.30	-0.39^∗^	-0.23	0.52^†^	0.27
IL-8	-0.06	-0.36^∗^	-0.30	-0.34^∗^	0.28	0.23
MCP1 (log)	-0.07	-0.24	-0.10	-0.25	0.10	0.20
MIP-1*α*	0.07	-0.07	-0.16	-0.20	0.23	0.22
PDGF (log)	0.25	-0.21	-0.10	-0.17	0.27	0.28
TNF*α* (log)	0.14	-0.31	-0.32^∗^	-0.38^∗^	0.46^‡^	0.40^∗^
VEGF-A (log)	-0.07	-0.28	-0.37^∗^	-0.28	0.32^∗^	0.14

^∗^ *p* ≤ 0.05, ^‡^ *p* < 0.01, and ^†^ *p* < 0.001. Data were analyzed using the Pearson test and reported as correlation coefficients *r*.

**Table 3:** Correlation pattern between intermediates in the arginine/NO pathway in patients with cardiometabolic diseases with and without chronic wounds.

Metabolite	Arginine	Citrulline	ADMA	SDMA (log)	Arg/ADMA	Arg/SDMA
Arginine	*—*	*-0.14*	*-0.15*	*0.23*	*—*	*—*
Citrulline	0.20	*—*	*0.81^†^*	*0.85^†^*	*-0.62^†^*	*-0.63^†^*
ADMA	0.45^†^	0.69^†^	*—*	*0.84^†^*	*—*	*—*
SDMA (log)	0.28^∗^	0.63^†^	0.68^†^	*—*	*—*	*—*
Arg/ADMA	*—*	-0.35^‡^	*—*	*—*	*—*	
Arg/SDMA	*—*	-0.36^‡^	*—*	*—*	*—*	*—*

The right side of the table (in italics) presents correlations in patients without wounds; the left side (in a straight script) presents correlations in patients with chronic wounds. ^∗^ *p* ≤ 0.05, ^‡^ *p* < 0.01, and ^†^ *p* < 0.001. Data were analyzed using the Pearson test and reported as correlation coefficients *r*.

## Data Availability

The raw data used to support the findings of this study are available from the corresponding author upon reasonable request.

## Supplementary Materials

Supplementary 1 Supplementary Table 1. Effect of sex on intermediates in the arginine/NO pathway among controls and patients with cardiometabolic burden without or with chronic wounds.

Supplementary 2 Supplementary Figure 1. The association of the gangrene presence with intermediates in the arginine/NO pathway.

Supplementary 3 Supplementary Figure 2. The association of the gangrene type with intermediates in the arginine/NO pathway.

Supplementary 4 Supplementary Figure 3. The association of the phlegmon presence with intermediates in the arginine/NO pathway.

Supplementary 5 Supplementary Figure 4. The association of ulcers with intermediates in the arginine/NO pathway.

Supplementary 6 Supplementary Figure 5. Difference in intermediates in the arginine/NO pathway between ulnar and femoral veins.
