# Supplementary material for: Metabolites of the Nitric Oxide (NO) Pathway Are Altered and Indicative of Reduced NO and Arginine Bioavailability in Patients with Cardiometabolic Diseases Complicated with Chronic Wounds of Lower Extremities: Targeted Metabolomics Approach (LC-MS/MS)
Source: Oxid Med Cell Longev. 2019 Jul 14;2019:5965721. doi: 10.1155/2019/5965721 (PMC6664544; doi:10.1155/2019/5965721)
Supplement: Supplementary 1 — Supplementary Table 1. Effect of sex on intermediates in the arginine/NO pathway among controls and patients with cardiometabolic burden without or with chronic wounds. [file 5965721.f1.docx]

“Metabolites of nitric oxide (NO) pathway are altered and indicative of reduced NO and arginine bioavailability in patients with cardiometabolic diseases complicated with chronic wounds of lower extremities – targeted metabolomics approach (LC-MS/MS)” by Krzystek-Korpacka et al.

**Supplementary Table 1:** Effect of sex on intermediates in arginine/NO pathway among controls and patients with cardiometabolic burden without or with chronic wounds.

| **Intermediate** | **Mean±SD or median (IQR) in females vs. males, *p* value** | | |
| --- | --- | --- | --- |
|  | **Healthy controls** | **Patients without wounds** | **Patients with wounds** |
| **Arginine [µM]** | 148.4±38.7 vs. 146.9±41.9, p=0.869 | 169.9±33.1 vs. 158.9±34.4, p=0.236 | 149.3±44.8 vs. 152.1±52.2, p=0.836 |
| **Citrulline [µM]** | 51.4±17.3 vs. 54.6±16.4, p=377 | 22.3 (18-26) vs. 38.9 (25-52), p<0.001 | 41.7±20.8 vs. 38.6±21.3, p=0.583 |
| **ADMA [µM]** | 0.404±0.07 vs. 0.398±0.07, p=0.704 | 0.38 (0.34-0.41) vs. 0.45 (0.37-0.67), p=0.007 | 0.67±0.19 vs. 0.644±0.2, p=0.640 |
| **SDMA [µM]** | 0.313±0.05 vs. 0.313±0.06, p=0.996 | 0.31 (0.27-0.33) vs. 0.39 (0.31-0.52), p=0.003 | 0.657±0.26 vs. 0.59±0.33, p=0.415 |
| **Arg/ADMA** | 380±130 vs. 380±130, p=0.985 | 421 (363-509) vs. 321 (248-466), p=0.030 | 231±71 vs. 247±85, p=0.458 |
| **Arg/SDMA** | 490±169 vs. 491±179, p=0.991 | 559±175 vs. 417±196, p=0.006 | 262±167 vs. 299±139, p=0.368 |

SD, standard deviation; IQR, interquartile range.
