# Supplementary material for: Metabolites of the Nitric Oxide (NO) Pathway Are Altered and Indicative of Reduced NO and Arginine Bioavailability in Patients with Cardiometabolic Diseases Complicated with Chronic Wounds of Lower Extremities: Targeted Metabolomics Approach (LC-MS/MS)
Source: Oxid Med Cell Longev. 2019 Jul 14;2019:5965721. doi: 10.1155/2019/5965721 (PMC6664544; doi:10.1155/2019/5965721)
Supplement: Supplementary 4 — Supplementary Figure 3. The association of the phlegmon presence with intermediates in the arginine/NO pathway. [file 5965721.f4.docx]

“Metabolites of nitric oxide (NO) pathway are altered and indicative of reduced NO and arginine bioavailability in patients with cardiometabolic diseases complicated with chronic wounds of lower extremities – targeted metabolomics approach (LC-MS/MS)” by Krzystek-Korpacka et al.

|  |  |
| --- | --- |
|  |  |
|  |  |

**Supplementary Figure 3:** The association of phlegmon presence with intermediates in arginine/NO pathway: **(a)** arginine; **(b)** citrulline; **(c)** SDMA; **(d)** arginine-to-ADMA ratio (Arg/ADMA); **(e)** arginine-to-SDMA ratio (Arg/SDMA). Data presented as means with 95% confidence intervals and analyzed using t-test for independent samples.
